# 1-(2-Carb­oxy­eth­yl)-3-(carboxyl­atometh­yl)-2-ethyl­benzimidazol-1-ium monohydrate

**DOI:** 10.1107/S1600536813006855

**Published:** 2013-03-16

**Authors:** Hong Yao, Yong-Qiang Xie, Xiao-Qiang Yao, Yun-Xia Yang, You-Ming Zhang

**Affiliations:** a Key Laboratory of Eco-Environment-Related Polymer Materials, Ministry of Education of China, Key Laboratory of Polymer Materials of Gansu Province, College of Chemistry and Chemical Engineering, Northwest Normal University, Lanzhou 730070, People’s Republic of China

## Abstract

In the title compound, C_14_H_16_N_2_O_4_·H_2_O, three substituent groups are located on the same side of the benzimidazole ring plane. In the crystal, O—H⋯O hydrogen bonds and π–π stacking between parallel imidazole rings [centroid–centroid distance = 3.858 (4) Å] link the mol­ecules into a three-dimensional supra­molecular structure.

## Related literature

For general background to supra­molecular coordination complexes, see: Chakrabarty *et al.* (2011 ▶); Cook *et al.* (2012 ▶); Wang *et al.* (2009 ▶, 2010 ▶). For related structures, see: Wei *et al.* (2012 ▶); Chen & Huang (2006 ▶); Wu *et al.* (2012 ▶).
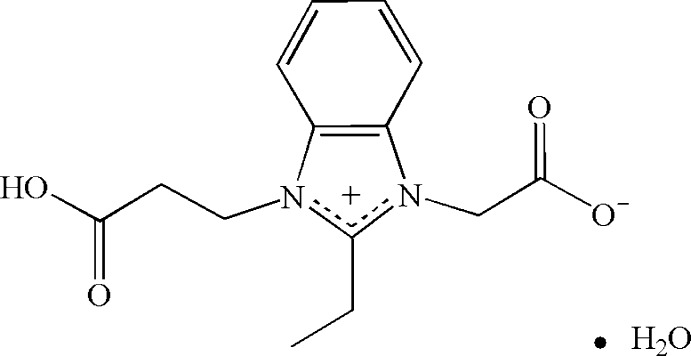


## Experimental

### 

#### Crystal data

C_14_H_16_N_2_O_4_·H_2_O
*M* *_r_* = 294.30
Triclinic, 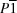

*a* = 8.286 (7) Å
*b* = 9.041 (8) Å
*c* = 10.629 (9) Å
α = 69.905 (7)°
β = 69.096 (7)°
γ = 79.082 (8)°
*V* = 696.6 (10) Å^3^
*Z* = 2
Mo *K*α radiation
μ = 0.11 mm^−1^
*T* = 296 K
0.26 × 0.23 × 0.22 mm

#### Data collection

Bruker APEXII CCD diffractometer
Absorption correction: multi-scan (*SADABS*; Bruker, 2001 ▶) *T*_min_ = 0.973, *T*_max_ = 0.977
5039 measured reflections
2551 independent reflections
1888 reflections with *I* > 2σ(*I*)
*R*_int_ = 0.022

#### Refinement

*R*[*F* ^2^ > 2σ(*F* ^2^)] = 0.042
*wR*(*F* ^2^) = 0.102
*S* = 1.07
2551 reflections
200 parameters
H atoms treated by a mixture of independent and constrained refinement
Δρ_max_ = 0.18 e Å^−3^
Δρ_min_ = −0.23 e Å^−3^

### 

Data collection: *APEX2* (Bruker, 2007 ▶); cell refinement: *SAINT* (Bruker, 2007 ▶); data reduction: *SAINT*; program(s) used to solve structure: *SHELXS97* (Sheldrick, 2008 ▶); program(s) used to refine structure: *SHELXL97* (Sheldrick, 2008 ▶); molecular graphics: *SHELXTL* (Sheldrick, 2008 ▶); software used to prepare material for publication: *SHELXTL*.

## Supplementary Material

Crystal structure: contains datablock(s) I, global. DOI: 10.1107/S1600536813006855/xu5687sup1.cif

Structure factors: contains datablock(s) I. DOI: 10.1107/S1600536813006855/xu5687Isup2.hkl

Supplementary material file. DOI: 10.1107/S1600536813006855/xu5687Isup3.cml

Additional supplementary materials:  crystallographic information; 3D view; checkCIF report

## Figures and Tables

**Table 1:** Hydrogen-bond geometry (Å, °)

*D*—H⋯*A*	*D*—H	H⋯*A*	*D*⋯*A*	*D*—H⋯*A*
O3—H3*A*⋯O1^i^	0.82	1.69	2.474 (2)	160
O5—H1*W*⋯O4^ii^	0.96 (3)	1.89 (3)	2.841 (3)	168 (2)
O5—H2*W*⋯O2^i^	0.90 (3)	1.96 (3)	2.851 (3)	176 (3)

Symmetry codes: (i) 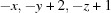; (ii) 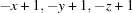.

**Fig. 1.**
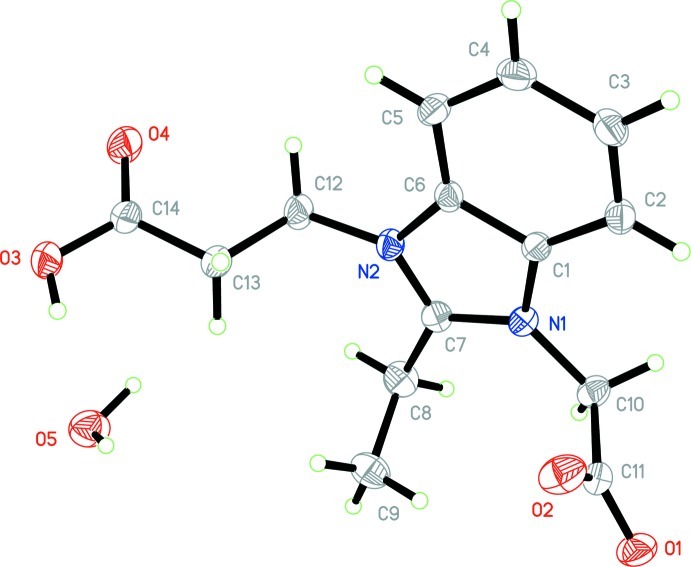
The molecular structure of the title compound with atom-numbering scheme. Displacement ellipsoids are drawn at the 30% probability level.

**Fig. 2.**
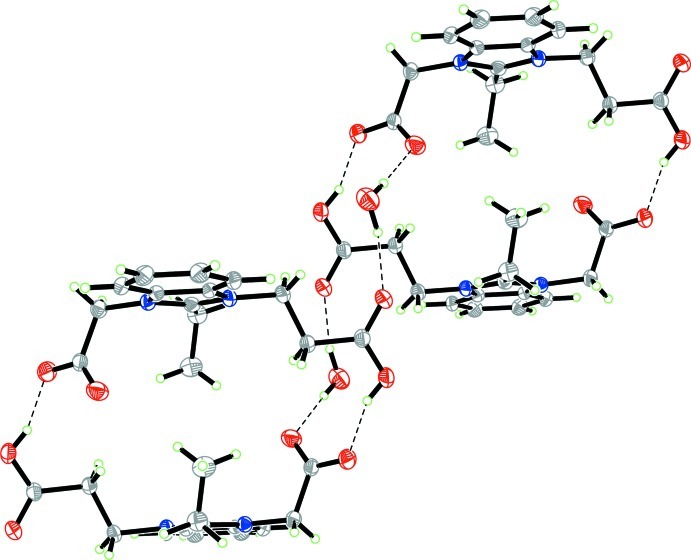
Hydrogen-bonded linking pattern of the wide hydrogen-bonded ribbon in the crystal structure of the title compound.

**Fig. 3.**
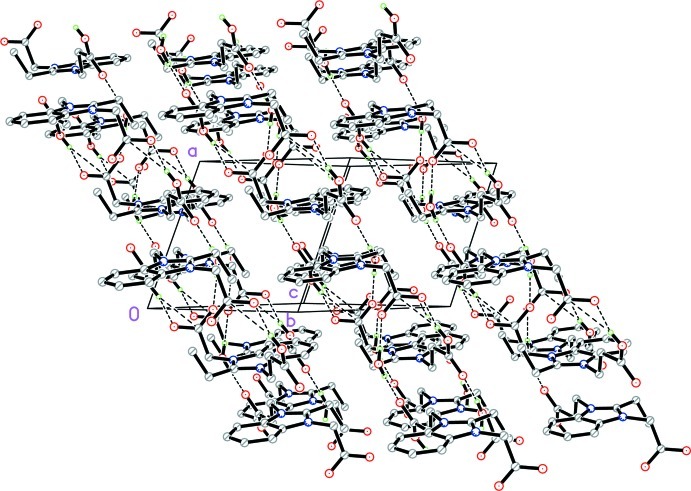
Packing diagram of the title compound. All hydrogen atoms bonded to carbon are omitted for clarity.

## supplementary crystallographic information

### Comment

Benzimidazole and its derivatives are strongly coordinating agents and form stable complexes with various metals, which find various application in supramolecular coordination complexes, catalytic systems and soft materials (Chakrabarty *et al.*, 2011; Cook *et al.*, 2012; Wang *et al.*, 2009; Wei *et al.*, 2012). Meanwhile, the coordination chemistry of dicarboxylates has gained great attention for a variety of reasons, such as good conformation freedom, various coordination modes and so on (Wang *et al.*, 2010; Wu *et al.*, 2012). Herein we report the crystal structure of 1-(2-acetoxy)-3-(3-propionyloxy)-2-ethyl-3*H*-benzimidazolium salt and the molecular structure is shown in Fig 1. In the imidazolium ring, the bond lenghs range from 1.338 (2) to 1.397 (2) Å, in good agreement with the presence of conjugated double bonds and indicating a zwitterionic structure (Chen & Huang, 2006). In the U-shaped molecule, the two carboxyl group run almost perpendicular to the benzimidazolium plane in the same orientation. The N1—C10—C11 and N2—C12—C13 angles are 112.57 (14) and 113.06 (15)°, respectively. A dimer is formed by benzimidazolium salt connected to neighboring molecule through O3—H3A ···O1 hydrogen bonds (Fig 2). In addition, the dimer makes full use of two water molecules as bridging molecules to form various O—H···O hydrogen bonds to generate the wide hydrogen-bond ribbon (Fig 3). Obviously, water molecules, as a kind of linking unit, play an important role in constructing this structure. The distance between the donor and acceptor of the hydrogen bond is in the range of 2.474 (2)–2.851 (3) Å. π-π stacking is observed between parallel imidazole rings of adjacent molecules [1-x, 2-y, -z], the centroids distance being 3.858 (4) Å.

### Experimental

1-(2-Acetoxy)-3-(3-propionyloxy)-2-ethyl-3*H*-benzimidazolium salt (0.2 mmol, 0.0552 g) was dissolved in distilled water. Colorless block crystals separated after several weeks.

### Refinement

Water H atoms were located in a difference Fourier map and refined isotropically. Other H atoms were positioned geometrically and refined as riding atoms, with C—H = 0.93 and O—H = 0.82 Å, *U*_iso_(H) = 1.5U_eq_(C,O).

### Crystal data

**Table:**

C_14_H_16_N_2_O_4_·H_2_O	*Z* = 2
*M**_r_* = 294.30	*F*(000) = 312
Triclinic, *P*1	*D*_x_ = 1.403 Mg m^−^^3^
Hall symbol: -P 1	Mo *K*α radiation, λ = 0.71073 Å
*a* = 8.286 (7) Å	Cell parameters from 1681 reflections
*b* = 9.041 (8) Å	θ = 2.4–26.1°
*c* = 10.629 (9) Å	µ = 0.11 mm^−^^1^
α = 69.905 (7)°	*T* = 296 K
β = 69.096 (7)°	Block, colourless
γ = 79.082 (8)°	0.26 × 0.23 × 0.22 mm
*V* = 696.6 (10) Å^3^	

### Data collection

**Table:**

Bruker APEXII CCD diffractometer	2551 independent reflections
Radiation source: fine-focus sealed tube	1888 reflections with *I* > 2σ(*I*)
Graphite monochromator	*R*_int_ = 0.022
φ and ω scans	θ_max_ = 25.5°, θ_min_ = 2.4°
Absorption correction: multi-scan (*SADABS*; Bruker, 2001)	*h* = −10→9
*T*_min_ = 0.973, *T*_max_ = 0.977	*k* = −10→10
5039 measured reflections	*l* = −12→12

### Refinement

**Table:**

Refinement on *F*^2^	Primary atom site location: structure-invariant direct methods
Least-squares matrix: full	Secondary atom site location: difference Fourier map
*R*[*F*^2^ > 2σ(*F*^2^)] = 0.042	Hydrogen site location: inferred from neighbouring sites
*wR*(*F*^2^) = 0.102	H atoms treated by a mixture of independent and constrained refinement
*S* = 1.07	*w* = 1/[σ^2^(*F*_o_^2^) + (0.0456*P*)^2^ + 0.0692*P*] where *P* = (*F*_o_^2^ + 2*F*_c_^2^)/3
2551 reflections	(Δ/σ)_max_ < 0.001
200 parameters	Δρ_max_ = 0.18 e Å^−^^3^
0 restraints	Δρ_min_ = −0.23 e Å^−^^3^

### Special details

**Table:**

Geometry. All e.s.d.'s (except the e.s.d. in the dihedral angle between two l.s. planes) are estimated using the full covariance matrix. The cell e.s.d.'s are taken into account individually in the estimation of e.s.d.'s in distances, angles and torsion angles; correlations between e.s.d.'s in cell parameters are only used when they are defined by crystal symmetry. An approximate (isotropic) treatment of cell e.s.d.'s is used for estimating e.s.d.'s involving l.s. planes.
Refinement. Refinement of *F*^2^ against ALL reflections. The weighted *R*-factor *wR* and goodness of fit *S* are based on *F*^2^, conventional *R*-factors *R* are based on *F*, with *F* set to zero for negative *F*^2^. The threshold expression of *F*^2^ > σ(*F*^2^) is used only for calculating *R*-factors(gt) *etc*. and is not relevant to the choice of reflections for refinement. *R*-factors based on *F*^2^ are statistically about twice as large as those based on *F*, and *R*- factors based on ALL data will be even larger.

### Fractional atomic coordinates and isotropic or equivalent isotropic displacement parameters (Å^2^)

**Table:**

	*x*	*y*	*z*	*U*_iso_*/*U*_eq_	
C1	0.2641 (2)	1.08245 (19)	0.06635 (18)	0.0295 (4)	
C2	0.2190 (2)	1.1649 (2)	−0.05422 (18)	0.0358 (4)	
H2	0.2043	1.2747	−0.0837	0.043*	
C3	0.1973 (2)	1.0757 (2)	−0.12773 (19)	0.0420 (5)	
H3	0.1676	1.1265	−0.2096	0.050*	
C4	0.2185 (3)	0.9111 (2)	−0.0833 (2)	0.0423 (5)	
H4	0.2019	0.8555	−0.1363	0.051*	
C5	0.2629 (2)	0.8285 (2)	0.03621 (19)	0.0388 (5)	
H5	0.2767	0.7188	0.0657	0.047*	
C6	0.2861 (2)	0.91826 (19)	0.11035 (17)	0.0309 (4)	
C7	0.3413 (2)	1.0071 (2)	0.26096 (19)	0.0333 (4)	
C8	0.3829 (3)	1.0131 (2)	0.3834 (2)	0.0455 (5)	
H8A	0.4589	1.0970	0.3522	0.055*	
H8B	0.4449	0.9142	0.4195	0.055*	
C9	0.2221 (3)	1.0414 (3)	0.5024 (2)	0.0526 (6)	
H9A	0.1572	1.1366	0.4663	0.079*	
H9B	0.2565	1.0516	0.5760	0.079*	
H9C	0.1513	0.9539	0.5396	0.079*	
C10	0.2867 (2)	1.2987 (2)	0.1588 (2)	0.0375 (5)	
H10A	0.3267	1.3640	0.0613	0.045*	
H10B	0.3618	1.3096	0.2067	0.045*	
C11	0.1029 (3)	1.3575 (2)	0.22808 (19)	0.0371 (4)	
C12	0.3833 (2)	0.7124 (2)	0.30663 (19)	0.0417 (5)	
H12A	0.4830	0.7123	0.3342	0.050*	
H12B	0.4172	0.6495	0.2422	0.050*	
C13	0.2397 (2)	0.63694 (19)	0.43592 (19)	0.0370 (4)	
H13A	0.2173	0.6898	0.5066	0.044*	
H13B	0.1347	0.6500	0.4113	0.044*	
C14	0.2846 (3)	0.4640 (2)	0.4969 (2)	0.0388 (5)	
N1	0.29992 (18)	1.13350 (15)	0.16246 (15)	0.0312 (4)	
N2	0.33392 (18)	0.87598 (16)	0.23231 (15)	0.0334 (4)	
O1	0.09505 (19)	1.48021 (15)	0.26178 (15)	0.0534 (4)	
O2	−0.01868 (18)	1.28600 (16)	0.24520 (16)	0.0548 (4)	
O3	0.1787 (2)	0.38995 (15)	0.61738 (15)	0.0562 (4)	
H3A	0.0996	0.4523	0.6453	0.084*	
O4	0.41378 (18)	0.39251 (15)	0.43815 (15)	0.0523 (4)	
O5	0.3224 (3)	0.5657 (2)	0.8276 (2)	0.0651 (5)	
H1W	0.405 (4)	0.594 (3)	0.734 (3)	0.097 (10)*	
H2W	0.230 (4)	0.614 (4)	0.800 (3)	0.114 (12)*	

### Atomic displacement parameters (Å^2^)

**Table:**

	*U*^11^	*U*^22^	*U*^33^	*U*^12^	*U*^13^	*U*^23^
C1	0.0273 (10)	0.0276 (9)	0.0312 (9)	−0.0028 (7)	−0.0062 (7)	−0.0088 (7)
C2	0.0375 (11)	0.0312 (9)	0.0334 (10)	−0.0035 (8)	−0.0093 (8)	−0.0047 (8)
C3	0.0484 (13)	0.0456 (11)	0.0298 (10)	−0.0060 (9)	−0.0126 (9)	−0.0072 (8)
C4	0.0490 (13)	0.0449 (11)	0.0365 (11)	−0.0089 (9)	−0.0086 (9)	−0.0187 (9)
C5	0.0426 (11)	0.0280 (9)	0.0412 (11)	−0.0042 (8)	−0.0047 (9)	−0.0129 (8)
C6	0.0280 (10)	0.0293 (9)	0.0294 (9)	−0.0021 (7)	−0.0054 (7)	−0.0055 (7)
C7	0.0268 (10)	0.0361 (10)	0.0349 (10)	0.0004 (7)	−0.0099 (8)	−0.0094 (8)
C8	0.0450 (12)	0.0533 (12)	0.0434 (11)	−0.0027 (9)	−0.0218 (10)	−0.0133 (9)
C9	0.0569 (14)	0.0669 (14)	0.0397 (11)	−0.0077 (11)	−0.0166 (10)	−0.0200 (10)
C10	0.0400 (11)	0.0311 (9)	0.0450 (11)	−0.0064 (8)	−0.0130 (9)	−0.0141 (8)
C11	0.0427 (12)	0.0296 (10)	0.0349 (10)	−0.0028 (9)	−0.0103 (9)	−0.0069 (8)
C12	0.0397 (11)	0.0323 (10)	0.0416 (11)	0.0075 (8)	−0.0123 (9)	−0.0030 (8)
C13	0.0397 (11)	0.0306 (10)	0.0382 (10)	0.0002 (8)	−0.0121 (9)	−0.0090 (8)
C14	0.0432 (12)	0.0324 (10)	0.0389 (11)	0.0001 (9)	−0.0140 (9)	−0.0091 (8)
N1	0.0322 (8)	0.0284 (8)	0.0343 (8)	−0.0022 (6)	−0.0113 (7)	−0.0101 (6)
N2	0.0350 (9)	0.0286 (8)	0.0322 (8)	0.0015 (6)	−0.0116 (7)	−0.0050 (6)
O1	0.0616 (10)	0.0376 (8)	0.0585 (9)	−0.0083 (7)	−0.0041 (7)	−0.0246 (7)
O2	0.0385 (9)	0.0531 (9)	0.0754 (11)	−0.0040 (7)	−0.0103 (7)	−0.0303 (8)
O3	0.0669 (11)	0.0318 (7)	0.0469 (8)	0.0043 (7)	−0.0031 (8)	−0.0036 (6)
O4	0.0472 (9)	0.0376 (8)	0.0580 (9)	0.0093 (7)	−0.0098 (7)	−0.0111 (7)
O5	0.0661 (12)	0.0636 (11)	0.0644 (12)	0.0109 (9)	−0.0241 (11)	−0.0227 (9)

### Geometric parameters (Å, º)

**Table:**

C1—C2	1.385 (3)	C9—H9C	0.9600
C1—N1	1.392 (2)	C10—N1	1.464 (2)
C1—C6	1.392 (3)	C10—C11	1.518 (3)
C2—C3	1.372 (3)	C10—H10A	0.9700
C2—H2	0.9300	C10—H10B	0.9700
C3—C4	1.395 (3)	C11—O2	1.225 (2)
C3—H3	0.9300	C11—O1	1.262 (2)
C4—C5	1.373 (3)	C12—N2	1.475 (2)
C4—H4	0.9300	C12—C13	1.505 (3)
C5—C6	1.387 (3)	C12—H12A	0.9700
C5—H5	0.9300	C12—H12B	0.9700
C6—N2	1.397 (2)	C13—C14	1.503 (3)
C7—N2	1.338 (2)	C13—H13A	0.9700
C7—N1	1.344 (2)	C13—H13B	0.9700
C7—C8	1.481 (3)	C14—O4	1.221 (2)
C8—C9	1.527 (3)	C14—O3	1.300 (2)
C8—H8A	0.9700	O3—H3A	0.8200
C8—H8B	0.9700	O5—H1W	0.96 (3)
C9—H9A	0.9600	O5—H2W	0.90 (3)
C9—H9B	0.9600		
			
C2—C1—N1	131.62 (16)	N1—C10—C11	112.57 (14)
C2—C1—C6	121.81 (16)	N1—C10—H10A	109.1
N1—C1—C6	106.55 (15)	C11—C10—H10A	109.1
C3—C2—C1	116.25 (17)	N1—C10—H10B	109.1
C3—C2—H2	121.9	C11—C10—H10B	109.1
C1—C2—H2	121.9	H10A—C10—H10B	107.8
C2—C3—C4	121.97 (18)	O2—C11—O1	127.11 (18)
C2—C3—H3	119.0	O2—C11—C10	119.49 (17)
C4—C3—H3	119.0	O1—C11—C10	113.39 (17)
C5—C4—C3	122.09 (18)	N2—C12—C13	113.06 (15)
C5—C4—H4	119.0	N2—C12—H12A	109.0
C3—C4—H4	119.0	C13—C12—H12A	109.0
C4—C5—C6	116.14 (17)	N2—C12—H12B	109.0
C4—C5—H5	121.9	C13—C12—H12B	109.0
C6—C5—H5	121.9	H12A—C12—H12B	107.8
C5—C6—C1	121.74 (17)	C14—C13—C12	111.66 (16)
C5—C6—N2	131.85 (16)	C14—C13—H13A	109.3
C1—C6—N2	106.40 (15)	C12—C13—H13A	109.3
N2—C7—N1	108.99 (16)	C14—C13—H13B	109.3
N2—C7—C8	125.90 (16)	C12—C13—H13B	109.3
N1—C7—C8	125.09 (17)	H13A—C13—H13B	107.9
C7—C8—C9	112.83 (17)	O4—C14—O3	119.89 (17)
C7—C8—H8A	109.0	O4—C14—C13	122.94 (17)
C9—C8—H8A	109.0	O3—C14—C13	117.17 (17)
C7—C8—H8B	109.0	C7—N1—C1	109.03 (15)
C9—C8—H8B	109.0	C7—N1—C10	126.07 (16)
H8A—C8—H8B	107.8	C1—N1—C10	124.85 (14)
C8—C9—H9A	109.5	C7—N2—C6	109.03 (14)
C8—C9—H9B	109.5	C7—N2—C12	126.86 (16)
H9A—C9—H9B	109.5	C6—N2—C12	123.88 (15)
C8—C9—H9C	109.5	C14—O3—H3A	109.5
H9A—C9—H9C	109.5	H1W—O5—H2W	95 (2)
H9B—C9—H9C	109.5		
			
N1—C1—C2—C3	178.24 (17)	C8—C7—N1—C1	−178.25 (16)
C6—C1—C2—C3	0.0 (3)	N2—C7—N1—C10	177.63 (15)
C1—C2—C3—C4	0.3 (3)	C8—C7—N1—C10	−0.7 (3)
C2—C3—C4—C5	−0.3 (3)	C2—C1—N1—C7	−178.66 (18)
C3—C4—C5—C6	−0.2 (3)	C6—C1—N1—C7	−0.20 (18)
C4—C5—C6—C1	0.5 (3)	C2—C1—N1—C10	3.8 (3)
C4—C5—C6—N2	−178.59 (18)	C6—C1—N1—C10	−177.77 (15)
C2—C1—C6—C5	−0.4 (3)	C11—C10—N1—C7	−94.1 (2)
N1—C1—C6—C5	−179.05 (15)	C11—C10—N1—C1	83.0 (2)
C2—C1—C6—N2	178.87 (16)	N1—C7—N2—C6	0.06 (19)
N1—C1—C6—N2	0.23 (17)	C8—C7—N2—C6	178.38 (16)
N2—C7—C8—C9	−100.2 (2)	N1—C7—N2—C12	174.64 (15)
N1—C7—C8—C9	77.8 (2)	C8—C7—N2—C12	−7.0 (3)
N1—C10—C11—O2	−19.1 (2)	C5—C6—N2—C7	178.99 (18)
N1—C10—C11—O1	161.19 (15)	C1—C6—N2—C7	−0.18 (19)
N2—C12—C13—C14	171.70 (16)	C5—C6—N2—C12	4.2 (3)
C12—C13—C14—O4	−7.5 (3)	C1—C6—N2—C12	−174.96 (15)
C12—C13—C14—O3	172.71 (17)	C13—C12—N2—C7	85.2 (2)
N2—C7—N1—C1	0.09 (19)	C13—C12—N2—C6	−101.0 (2)

### Hydrogen-bond geometry (Å, º)

**Table:**

*D*—H···*A*	*D*—H	H···*A*	*D*···*A*	*D*—H···*A*
O3—H3*A*···O1^i^	0.82	1.69	2.474 (2)	160
O5—H1*W*···O4^ii^	0.96 (3)	1.89 (3)	2.841 (3)	168 (2)
O5—H2*W*···O2^i^	0.90 (3)	1.96 (3)	2.851 (3)	176 (3)

Symmetry codes: (i) −*x*, −*y*+2, −*z*+1; (ii) −*x*+1, −*y*+1, −*z*+1.
